# Hints upon the Extraction of Teeth

**Published:** 1855-04

**Authors:** 


### ARTICLE VIII.

### *Hints upon the Extraction of Teeth.*

Few operations, that a dentist is called upon to perform, are attended with so many unpleasant consequences as the extraction of teeth, not excepting the insertion of *unpaid* mechanical work. We may divide teeth for removal into three classes, as regards their difficulty, first, those which, from the nature of their growth, require little or no skill in removing; second, those which require a little skill and much strength; third, those which require great skill with much force. Every tooth should be both *luxated* and *rotated*, without exception, for the purpose of breaking up their attachment in the socket, and for the purpose of forcing the walls of the socket away from the fangs; a mere luxative movement, as is so generally adopted by operators, can only produce these objects in two directions, or in two parts, instead of the whole, leaving much the larger half unmolested. Again, by a rotatory movement there will be often discovered an inclination that the force should be made in some exact direction, in which the tooth will be most readily removed, by accommodating some peculiar growth of the fangs, or by the appliance of the power in the axis of the growth of the tooth, which would often remain entirely obscure when a mere in and out motion were made.

The first class we spoke of requires but little description or comment, except that their removal should invariably be performed without haste, and with a due deliberation, to prevent fracture and unnecessary pain, which is always produced when a violent and sudden force compels the separation of sensitive parts; the fracturing of teeth, so ruinous to a practitioner's reputation, is, in nine cases out of ten, produced by this misapplied animal force, and as an infallible consequence, the patient made to bear a cruel and unnecessary torture, or exposed to all the morbid effects of a dead and irritative fang left in an injured alveolus, surrounded by an inflamed gum. This class generally occupy a vertical position in their sockets, imbedded in very soft and elastic alveoli, with a thick periosteum, permiting an easy egress of the organs. They are found in gums diseased by constitutional causes, and consequently flabby, and suffering perpetually with chronic inflammation, which, by connection, changes the nature and office of the periosteum, and the teeth in such cases are generally extracted almost as easy as beans are from a pod. Teeth whose fangs are poorly developed and small, wide spaces existing between them—teeth which have afforded means of attachment for artificial work, or pivot teeth—these are all removed without requiring skill, or any great amount of practice—a mere bold confidence often being the only thing required to perform wonders to the patient.

The second are those which belong to a better constitution, or one in whom primitive good health has been unimpaired to any great extent, or at least during the time the osseous system is being fully perfected. The teeth in this case are finely developed and firmly set, tapering from the neck to the grinding surface, large alveoli, covered with a firm gum, possessed by individuals whose muscular energy is present in every action, having a sanguino-nervous temperament. The extraction of such teeth will require some skill and considerable power, carefully directed, particularly, if their relative growth is irregular in the alveolus, or when the lines of their axes would meet at an acute angle, some two inches above their necks; in which case the rotary motion is peculiarly good, for it enables an operator to detect this condition of affairs at once, and compels him to apply the extractive force in the proper direction, instead of against the neighboring tooth, as would be the case, most likely, where mere luxation is used. Often whole sets of teeth are found presenting the line of their axes running from the front part backwards, requiring the instrument to be forced back against the lips, so as to apply the pressure in the proper line. All such teeth to be removed safely and with skill, must be removed slowly and without haste—being one of those operations which when done well, is freed from any great amount of excessive pain, but when badly and hastily performed, may be attended not only with agony, but with a very formidable danger. Every operator of any experience will recognize the truth of these remarks, and no doubt call to mind much success in accomplishing by delicate efforts that which had been abandoned by those who operate in haste and excitement. It is well, too, that a patient should fully appreciate the difficulties of all serious cases of extraction, so that you may be properly rewarded for the completion of an operation, which presented great and almost insurmountable difficulties. In looking over my case-book I find recorded the circumstances of a case of extraction, which clearly proves the necessity of a patient's entire comprehension of an operator's applied skill. The person was a most remarkably well made man, of about thirty-five years, a farmer and stone-cutter; every muscle was fully developed, and most actively rounded; possessed of much firmness and resolution. He had suffered for some weeks from two teeth, one of which the disease had exposed the pulp cavity; the other had not progressed quite so far, yet badly diseased. He had the attempt to extract them made by three different dentists, and some physicians, but without the least success or relief. He had travelled through the country sixty miles on horse-back to apply to me, determined to get relief if at all possible. I found that the great obstacle to their tolerably easy removal resided in the anterior and posterior tooth pressing against and holding them all together by a peculiar interlocked growth, and at the same time were large, well made, and fixed firmly in an alveolus more than usually dense. I determined to extract the one, fill the other, and, as was the custom, the price agreed upon at once. I used a sharp file, which made a free separation on either side of the tooth down a slight distance below the gum—this was a left inferior molar, and to make more sure of success, I used a strong sharp pointed instrument to lift the alveolus away from the tooth about the junction of the crown and fangs, so that the points of the forceps should readily enter between the fangs and grasp the tooth thoroughly. It came with ease and certainty, but after the pain had subsided he objected to pay the price we had absolutely agreed upon for the operation.

The third class is to be found in persons of intense strength of muscle, whose bones possess the greatest density, and are generally formed of the medium size, but who are possessed of all energy, motion, and activity—whose teeth may not be so firmly interlocked, but still in close contact, so as to prevent any motion from the tooth of removal being communicated to adjoining teeth, and require the free use of the file; but the great difficulty resides in the steel-like resistance of the bone of the socket, in connection with the bifurcation of the fangs—this can be only overcome by the lifting off the alveolus from around the tooth, after it has been separated in a free manner; then with a rotary motion, combined with luxation, any tooth can be removed with certainty and safety, provided, time and care has been taken in applying the force for its removal. A. A. B.

